# RETRACTED: Atta et al. Synthesis of Environmentally Friendly Highly Dispersed Magnetite Nanoparticles Based on Rosin Cationic Surfactants as Thin Film Coatings of Steel. *Int. J. Mol. Sci.* 2014, *15*, 6974–6989

**DOI:** 10.3390/ijms27010011

**Published:** 2025-12-19

**Authors:** Ayman M. Atta, Gamal A. El-Mahdy, Hamad A. Al-Lohedan, Sami A. Al-Hussain

**Affiliations:** 1 Surfactant Research Chair, Chemistry Department, College of Science, King Saud University, P.O. Box-2455, Riyadh 11451, Saudi Arabia; khaled_00atta@yahoo.com (A.M.A.); hlohedan@ksu.edu.sa (H.A.A.-L.); imamchemistry@gmail.com (S.A.A.-H.); 2 Petroleum Application Department, Egyptian Petroleum Research Institute, Cairo 11795, Egypt; 3 Chemistry Department, Faculty of Science, Helwan University, Helwan, Cairo 11727, Egypt

The journal retracts the article “Synthesis of Environmentally Friendly Highly Dispersed Magnetite Nanoparticles Based on Rosin Cationic Surfactants as Thin Film Coatings of Steel” [1] cited above.

Following publication, concerns were brought to the attention of the Editorial Office regarding the integrity of an image presented in this article [1].

Adhering to our standard procedure, an investigation was conducted by the Editorial Office and Editorial Board that identified indications of inappropriate editing in Figures 1, 2 and 4a presented in this article [1]. While the authors cooperated with the Editorial Office during the investigation, they were unable to satisfactorily explain the identified issues and could not provide appropriate raw material meeting the journal’s requirements for original images (https://www.mdpi.com/journal/ijms/instructions#oriimages) for Editorial Board evaluation. Consequently, the Editorial Board has lost confidence in the reliability of the findings and decided to retract this publication [1], as per MDPI’s retraction policy (https://www.mdpi.com/ethics#_bookmark30).

This retraction was approved by the Editor-in-Chief of the journal *IJMS*.

The authors did not agree to this retraction.
